# A Review of Insect Monitoring Approaches with Special Reference to Radar Techniques

**DOI:** 10.3390/s21041474

**Published:** 2021-02-20

**Authors:** Alexey Noskov, Joerg Bendix, Nicolas Friess

**Affiliations:** Faculty of Geography, Philipps University of Marburg, Deutschhausstraße 12, 35032 Marburg, Germany; bendix@geo.uni-marburg.de (J.B.); nicolas.friess@geo.uni-marburg.de (N.F.)

**Keywords:** insect radar, conservation, remote sensing, light trap, FMCW radar, UGV

## Abstract

Drastic declines in insect populations are a vital concern worldwide. Despite widespread insect monitoring, the significant gaps in the literature must be addressed. Future monitoring techniques must be systematic and global. Advanced technologies and computer solutions are needed. We provide here a review of relevant works to show the high potential for solving the aforementioned problems. Major historical and modern methods of insect monitoring are considered. All major radar solutions are carefully reviewed. Insect monitoring with radar is a well established technique, but it is still a fast-growing topic. The paper provides an updated classification of insect radar sets. Three main groups of insect radar solutions are distinguished: scanning, vertical-looking, and harmonic. Pulsed radar sets are utilized for all three groups, while frequency-modulated continuous-wave (FMCW) systems are applied only for vertical-looking and harmonic insect radar solutions. This work proves the high potential of radar entomology based on the growing research interest, along with the emerging novel setups, compact devices, and data processing approaches. The review exposes promising insect monitoring solutions using compact radar instruments. The proposed compact and resource-effective setups can be very beneficial for systematic insect monitoring.

## 1. Introduction

The massive use of pesticides, intensive land-use, and climate change are sources of the damaging impact on nature, especially insects [1,2,3,4]. Insect conservation attracts significant worldwide interest in this context [5,6,7,8,9,10,11]. It is motivated by the human-caused global impact, which has resulted in the most remarkable episode of mass extinction since the loss of the dinosaurs 65 million years ago. In the scope of conservation biology, this extinction has become more recognizable with recent technological progress.

Researchers warn about the possibility of the extinction of 40% of insects over the next few decades [12]. About 400 insect species have recently been officially classed as extinct; some estimate that from 1 to 100 invertebrates species vanish every day [13]. This problem requires urgent attention not only because of insects’ usefulness as pollinators, pest control, and nutrient cycling agents but, mainly, because of their right to exist. The manifesto “World Scientists’ Warning to Humanity” (issued by the Alliance of World Scientists) details the leading factors that cause insect extinction: habitat loss, degradation, and fragmentation, the use of polluting and harmful substances, the spread of invasive species, global climate change, direct overexploitation, and the co-extinction of species dependent on other species [14]. It appeals for urgent action to close the critical knowledge gaps and curb insect extinction. Moreover, researchers expect that many pest species (e.g., houseflies and cockroaches) are likely to boom; it is formulated as a “pests on the rise” problem [12].

While the significant decline in insect numbers (often called “Ecological Armageddon”) is apparent and confirmed by many studies [15,16], researchers notice significant research gaps [17]. They emphasize that such works consider mainly biomass (not species). Moreover, sites are not sampled continuously and represent only limited areas. Didham et al. [18] have distinguished seven critical challenges in the interpretation of insect decline: the establishment of the historical baseline, the representativeness of site selection, the robustness of time series trend estimation, the mitigation of detection bias effects, and the ability to account for potential artifacts of density dependence, phenological shifts, and scale dependence in extrapolation from sample abundance to population-level inference. They have distinguished several opportunities for more creative exploitation of existing baseline data and have proposed to adopt the latest technological advances in sampling and novel computational approaches. Addressing the mentioned gaps and challenges requires significant additional resources. Recent achievements in technologies and automatizing allows researchers to increase the effectiveness of the resource use and, more importantly, move the research to a principally new level.

Today, cutting-edge works on insect monitoring mainly utilize non-destructive technical solutions on the market, such as automatic traps with novel sensors and machine learning systems. In Reference [19], Lima et al. discuss pest management automatic systems utilizing pest identification methods based on infrared sensors, audio sensors, and image-based classification. They presented the different systems available, examples of applications, and recent developments, including machine learning and the Internet of Things. Among recent relevant works on technically advanced insect monitoring, we want to distinguish research on small insects’ vertical motion using millimeter-wavelength radar and Doppler lidar for insect pest detection [20], migration, and monitoring. Radar and lidar systems also played a vital role in another recent article on insect pest detection and migration monitoring [21].

The earlier discussion indicates the high demand for systematic and global solutions. Among them, we can securely distinguish highly popular, but still enormously growing, entomology radar systems. This paper aims to review primary insect monitoring techniques with the main focus on radar technology for entomology purposes. Finally, we propose novel solutions and recommendations addressing the indicated gaps for future technical solutions and research.

## 2. Insect Monitoring Methods

It is a well-known fact that insects play a crucial role in ecosystems [22]. Being small and active, they remain almost invisible for most observers. A deeper understanding of ecosystems’ functionality requires a systematic monitoring of insects, including state-of-the-art and novel solutions. As mentioned earlier, the current monitoring still has significant gaps that have to be addressed. This section considers promising solutions in terms of automatization, effectiveness, and cost, thus their having widespread potential.

Medeiros et al. noticed that “insects receive low levels of conservation funding, likely due to their small size, fluctuating population sizes, and lack of baseline data necessary to determine if they are threatened with extinction” [23]. They proposed broad insect monitoring strategies focused on community-based metrics instead of individual species. That work considered a situation in Hawaii, where insects play a crucial role as pollinators [24], the primary native consumers of plants (due to the lack of herbivorous mammals), critical sources of protein for all native forest birds [25], and essential parts of food webs [26]. They mentioned the most apparent insect conservation difficulty: insects’ tiny size and cryptic life; it is often noticed only after careful observation of an ecosystem. Moreover, “many insects are nocturnal, or spend all or most of their lives under rocks, high in the tree canopy, or buried in moss or leaf litter” [23]. The authors noticed the limited capacity of modern GPS and radio technologies for insect monitoring (excluding the largest insects).

Due to the lack of information, monitoring should begin from community considerations and accurate assessments of population or species trends for at least a subset of the native fauna. Continuous collecting and monitoring efforts require the harvesting of critical information for synthesizing abundance and distribution of invertebrate species [27]. Basset et al. [28] indicated that broad sampling is the best current monitoring approach until such datasets can be used to determine whether a smaller subset of taxonomic or functional groups can be used. Despite this, in some specific cases, monitoring focusing on single species is required [29].

Moving down from general recommendations to concrete implementations, one can distinguish an increasing number of pest control works concerning insect monitoring. The apparent reason for this is that such insects cause the loss of billions of dollars and 14–15% of the world’s crops [30], which motivates the investments to the required research. In contrast to conservation purposes, concrete results applicable in practice are significant for pest control. As a result, such works are aimed at mathematical modeling and practical recommendations; both are very worth utilizing in the conservation realm.

Petrovskii et al. [31] provide an overview of the state-of-the-art mathematical approaches to insect monitoring for pest control, mainly focusing on random walks, pattern formation, synchronization, and networks. They distinguish two essential monitoring components: data collection and data processing or interpretation. They noticed that trapping is a standard working method in such work; it enables researchers to estimate the population density of harmful species at the traps’ positions. The authors considered three scale levels: a single trap, a single agricultural field comprising several traps, and several fields with multiple traps. Calculations on the second scale typically utilize the arithmetic average of local densities, but, as discussed, it becomes ineffective and inaccurate if the distribution is heterogeneous. Long-distance cross-correlations between the pest abundance in different fields are called synchronization. Inter-habitat dispersal and the effect of spatially correlated noise are two main synchronization mechanisms. The authors noticed that dispersal between different habitats could occur through a particular self-organized network, which can arise due to the interplay between environmental properties. Hence, close fields can be uncorrelated, but some distant fields can be almost perfectly synchronized. A type of trap plays a crucial role here. For instance, a trap can be a simple ground hole with a bowl or a baited trap. The rule of thumb for a trap size says that it is larger by a factor of ∼$10^{2}$.

**Figure 1:**
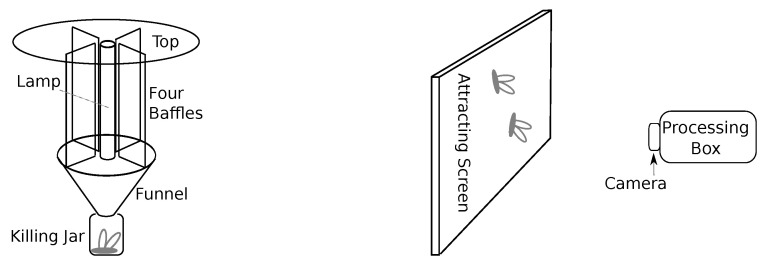
Evolution of light trap proven constructions from a classical schema of the Pennsylvania light trap (**left**) to typical construction of modern screen-camera light traps (**right**).

Insect trapping and, in particular, light trapping are primary methods for insect monitoring with a long history and a large corpus of relevant research publications and reviews [32,33,34,35,36,37,38,39]. These works confirm the effectiveness of trapping methods compared to other methods (e.g., sweeping). Moreover, they show the efficiency and high potential of light traps. Light traps remain a trending topic leveraging by recent advances in technology. Figure 1 depicts major proven light trap designs.

Today, researchers often combine light traps with cameras and use computer vision, pattern recognition, artificial intelligence, and machine learning algorithms for data processing. Due to the high economic demand, many such works aim to pest control. In this light, we want to notice the following relevant works on pest control for different agriculture types: vegetable [40,41], grass field [42,43], grain [44,45], and orchard [46,47]. The mentioned works motivated a paper published by Qing et al. [48]. They developed a light trap using several light sources, a glass screen, and two cameras. The authors extracted features from images for each rice pest; then, a support vector machine classifier with a radial basis kernel function was applied for identifying four species of Lepidoptera rice pests.

In Reference [49], Shimoda and Honda discussed insect reactions to light in the pest management context. That work comprises significant recent findings that are important to researchers working with light traps for insect monitoring. They emphasized that insects can see the visible light wavelength, which often expands into the ultraviolet (UV) range invisible to humans. For instance, honeybees’ compound eyes contain three types of photoreceptor cells with spectral sensitivity peaking in the UV, blue, and green wavelength ranges. Even though insects have variable light perception, and some are sensitive to red [50], we can securely say that many insects perceive UV light as a unique color [51,52]. Shimoda and Honda distinguished several types of insect reaction to artificial manipulations with the light (phototactic behaviors): attraction or moving toward a light source [53,54], repulsion or moving away from light [55,56], light adaptation or provoking daytime behaviors, such as the cessation of movement and settling down [57,58], phase shift or affecting the timing of the diurnal/nocturnal behavior [59,60], photoperiodicity or preventing the onset of diapause [61,62], light toxicity or damaging an insect [63,64], making objects invisible for insects by blocking the UV light [65,66], and, finally, dorsal light reaction or the disturbing the normal orientation of flight light reflected from below [67,68]. Shimoda and Honda noticed the impact of light on insect behavior. It varies both qualitatively and quantitatively. It also depends on the light source (either light bulb or light-emitting diode (LED)) and material (i.e., light-reflecting plate) and demonstrated the usage of various technologies to control pests. They concluded that LED devices with various wavelengths have great potential in agricultural technology.

In modern light traps, all disclosed main principles work perfectly. White et al. [69] described low-cost solutions for constructing “old-school” light traps using modern materials and devices. They constructed effective light traps using low-wavelength LED, soda bottles, funnels, and rechargeable 9-V batteries. Another feature of modern light traps is that researchers often combine them with cameras. Today, computer vision and machine learning facilitate insect detection from images taken with such cameras. Modern screen-based constructions are compact and approaching mass production [48]. Bjerge et al. [70] presented a recent example of such a construction. The authors apply a computer vision system to detect and classify live moths (Lepidoptera) using tracking and deep learning. The following elements facilitate effective moth capturing:

- a UV fluorescent tube,
- a light table (white screen, sometimes sprinkled with sugar water),
- a light ring (to ensure a diffuse foreground illumination of the insects),
- a web camera,
- a Raspberry Pi 4 computer configured for computer vision software, and
- a motion detection system allowing effective resource spending.

The techniques metioned before utilize a typical construction of modern light traps shown in Figure 1 right. Such screen-based systems represent the most simple solutions used with cameras. In contrast to the “old-school” solutions described earlier (see Figure 1 left), novel systems have one clear disadvantage: they illuminate the light in one direction (against the screen), which can lead to (according to the earlier mentioned findings) a decrease in caught insects. This paper presents a possible solution for this problem; our light trap prototype can illuminate the light in all directions.

Remote sensing approaches for insect monitoring are an alternative to sampling methods. Non-intrusive solutions with cameras for insect capturing are the closest remote sensing method to the earlier discussed systems. For instance, Ruczyński et al. [71] described a solution with a sky-oriented camera for nocturnal insect monitoring. These cameras took pictures at 5-min intervals from sunset to sunrise, with only 10% of them containing insects (a median number of 2). In contrast to the light-trap solutions, some can notice that the described approach with sky-oriented cameras belongs to the remote sensing realm (as well as unmanned-aerial-vehicle (UAV) cameras [72,73]).

Researchers apply a broad spectrum of remote sensing solutions, which is not limited to RGB cameras. Many works use radar technology, discussed in the next section. Furthermore, lidar devices show promising potential in the high-resolution remote sensing entomology [74,75,76,77,78,79,80,81,82,83]. Among recent works on lidar entomology, we want to mention the two following articles [84,85]. The former discloses the high potential of lidar for insect monitoring; for verification, the authors utilized a light trap setup nearby and demonstrated the technology’s capability to distinguish different types of insects during flight and quantify their movements. The authors illuminated Anopheles malaria mosquitoes from different angles with linearly polarized near-infrared light; they concluded that light scattered by insect wings could be separated from light scattered by insect bodies due to the oscillatory wingbeats. In 2019, Brydegaard and Jansson [86] reviewed several similar works.

Although lidar can be considered as a subset of the radar realm, we firmly separate these technologies in the present article. In what follows, we do not consider lidar.

## 3. Insect Radar

Among all remote sensing methods, radar is the biggest and the most promising. Insect radar has a long proven history and a great potential to fill the mentioned gaps in state-of-the-art insect monitoring solutions. It allows systematic and global solutions that address the main challenges of insect monitoring discussed in the introduction. Despite the long history, insect radar remains a growing topic with multiple challenges and unsolved problems.

Insect radar has a history of about 70 years. It likely started from Crawford [87] and was supplemented by other prominent works focused on individual insect detection [88,89], the retrieval of size [90], wingbeat [91], density [92,93], airspeed-heading [92,94], and flight duration and range [91]. Early research achievements have been summarized by Riley in Reference [90]. Drake and Reynolds, in their well-known book on radar entomology [95], have presented a systematic overview of insect radar applications and solutions before 2012. Since major publications and milestones were extensively discussed earlier in the literature, we do not discuss them in detail.

Recent works continue to fill research gaps in equipment [96,97,98], reflectivity, and insects’ geometrical characteristics [99,100,101,102]. As shown earlier, radar applications focus on either biodiversity/conservation [103,104,105,106,107] or agriculture (economically important insects [108,109,110] and pest management [21,111,112,113,114,115,116]).

Many recent works focus on new radar cross-section (RCS) information processing methods. For instance, Kong et al. [117] have presented a systematic investigation on insect cross sections in lab conditions; they conducted work with multiple frequencies and insect species and concluded that the measurement results confirmed the established system’s effectiveness and accuracy. Another recent article [102] introduced an approach to insect mass estimation with support vector regression; the best-reached estimation accuracy of insect mass in lab conditions was 78%. Wang et al. [118] proposed a new detection method for maneuvering targets with small RCS; it was tested with many simulations and good field experiments. In Reference [119], Alzaabi demonstrated computational electromagnetic tools to predict radar scattering aerial insects’ (honeybees) characteristics and investigated cross-section dependencies on multiple frequencies, polarizations, and viewing angles.

Often, radar studies group radar by the following frequency bands [120]:

- L-band (1–2 GHz, not utilized by insect radar),
- S-band (2–4 GHz),
- C-band (4–8 GHz),
- X-band (8–12 GHz),
- Ku-band (12–18 GHz), and
- Ka-band (26–40 GHz).

One can notice that many recent works consider Ku-band radar. Hu et al. [121] analyzed RCS characteristics for data measured in the field by a Ku-band high-resolution insect radar. In Reference [122], researchers proposed a novel method for automatic wing-beat frequency extraction for a Ku-band insect radar system (this set enables the high-resolution full-polarized detection of insects, but it has not achieved automatic extraction of the wing-beat frequency before). Cai et al. proposed an insect density estimation approach [123] using insects’ traces and classification based on RCS and heights.

Various mathematical solutions are considered for the improvement of the radar detection ability. For instance, Hu et al. [124] proposed using the second-order polynomial approximation for the insects’ horizontal speed estimation for high resolution and full polarization radar set. In Reference [125], the same research group has studied the invariant target parameters of a small sample of insects and proposed two methods for body mass and length calculation. In Reference [126], Fang et al. showed that gamma distribution could fit the insect target’s RCS probability distribution and introduced an RCS feature-aided tracking algorithm to improve the insect target tracking accuracy.

As in earlier work, signal polarization plays a vital role in modern insect radar research. Mao et al. [127] discussed a novel fully polarimetric insect radar with high range resolution for nocturnal migration monitoring. Moreover, they showed a technique for the extraction of individual biological parameters from the echo signal. Distinguishing birds and insects remains an essential challenge [128]; in Reference [129], the authors applied a fuzzy logic algorithm to use the dual-polarization S-band radar exploiting mainly for meteorological purposes.

Another significant aspect of recent research is the growing access to compact radar devices. Zulkifli and Balleri [130] pointed out the urgent demand in nano-target-oriented (less than 5 cm in size) radar solutions (e.g., for nano-drone [131] detection or pest management). They have considered frequency-modulated continuous-wave (FMCW) radar as a solution because it “can provide short-range detection, with no blind range and very high resolution, at a relatively low cost.” The authors have designed and developed a 24 GHz FMCW radar using off-the-shelf components and showed its feasibility.

All mentioned direction of recent research on insect radar is especially significant in light of concerns regarding the insect abundance decline [132]. Satterfield et al. [133] suggested further research and policy priorities to investigate and protect insect migrations concerning the mobile insect population decline and behavioral shift. Similar concerns motivated Stepanian et al. [104] for their work on the aquatic insect abundance decline in North America using radar “at scales that have been previously impossible.” Moreover, radar confirmed that the pest migration is under the severe impact of climate change [134].

Earlier works on insect radar clearly distinguish the following major groups: pulsed scanning, vertical-looking, and harmonic radar. Moreover, pulsed and FMCW sets are distinguished. The majority of insect radar system was based on pulsed radar, but FMCW radar is becoming very popular today. Because of this fact, we pay special attention to FMCW systems.

Further, we consider the main groups of insect radar and pay special attention to recent publications and aspects that have not been intensively discussed in the literature.

### 3.1. Pulsed Scanning Radar

The history of insect radar has been started from pulsed scanning radar solutions. Many notable works describe radar entomology’s achievements with scanning systems worldwide and discuss their main challenges [135,136,137,138,139,140,141].

Many recent articles aim to find solutions to filter out insects’ effect on weather radar data. For example, one of the concerns [142] is insects’ impact on Doppler radar wind observations. Classical threshold-based filtering remains a popular technique for this. Among novel solutions, machine learning approaches are becoming more popular [143]. Applying modern deep-learning algorithms enables the distinguishing of a tremendous amount of insect information in weather radar data [144,145]. Since a vast amount of weather radar data contaminated with biological echoes [128,146] is available, we can expect a very high trending interest in this topic in the future. This process is leveraged by advances in big data processing and machine learning progress.

Pulsed scanning weather radar remains a mainstream technology for insect monitoring using previous decades’ achievements and created radar networks [20,103,107,129,144,147,148,149]. This radar type has become a well developed solution.

### 3.2. Vertical-Looking Flight Observations

The vertical-looking radar (VLR) is another solution supplementing scanning systems and facilitating the distinguishing of insect species since it can provide higher-detail information (and, as a result, covers a much smaller area). Early vertical-looking radar sets have been introduced in the 1990s for entomology purposes [150,151], including the estimation of the population dynamics of migratory insect species [152,153], the impact of insect groups [154,155], and outbreaks of pest species [156]. Since 1996, researchers have started to apply the beam nutation [157] principle. It allows higher-precision altitudinal and temporal insect dynamics monitoring [158].

There is one interesting fact that has not been explicitly discussed in the literature. Observing papers on VLR published in the early 2000s, one can notice a “competition” between the English and Australian schools on insect radar. Researchers from the UK have actively promoted the “VLR” term, while Australian scientists have used an abbreviation for zenith-pointing linear-polarized conical-scan (ZLC). Moreover, Australian researchers proposed an abbreviation for their insect monitoring radar (IMR) and have actively used it in the following publications. In contrast to “VLR”, the “ZLC” and “IMR” abbreviations have not become popular outside the research group. In a collaborative review publication led by English researchers [159], the “VLR” term played a central role, while “ZLC” and “IMR” were not mentioned at all. In the later book [95] led by an Australian researcher, authors have actively used all three abbreviations. The next three paragraphs discuss the main milestones of this “competition.”

In Reference [160], Chapman et al. described a work conducted with a new VLR system. The device had a linearly polarized and slightly oscillated (0.18${}^{\circ }$ offset around the vertical axis) beam. The beam continuously rotated by mechanically turning the upward-pointing wave-guide feed about the vertical. It should be noticed that, in contrast to migrations observed with X-band scanning radar [161], vertical-looking radar can only define a group of migrating insects’ flight headings since it covers a small area. The heading is defined using the body orientation (alignment), displacement direction (however, the displacement direction is largely determined by the wind direction), and displacement speed of overflying migrants. Later, in Reference [162], the authors presented a systematic investigation of insect aerial density’s vertical distribution in the atmosphere for multiple temporal scales. They discussed, revealed by the radars, general features of insect stratification and occasions during warm nights in the summer months. The authors emphasized in the conclusions that nocturnal layering events are more important than it might seem from their frequency of occurrence because they often occur at night, and are particularly favorable to massive and sustained insect migrations.

Harman and Drake [163] published their approaches to VLR; they called it “zenith-pointing linear-polarized conical-scan (ZLC) configuration.” The author proposed “a relatively straightforward analytical method that uses the form of the ZLC scan to isolate signal components in the time-domain and that allows the parameters” (the target’s trajectory (speed and direction), its orientation, and its character (size and shape)) “to be estimated by a sequence of least-squares fits.” That was one of the first works where we encountered the “IMR” abbreviation for “Insect Monitoring Radar.” At that time, “VLR” and “ZLC”/“IMR” abbreviations applied in parallel to very close types of radar systems. Later, the “VLR” abbreviation has become commonly used in insect radar, while “ZLC” is not popular today.

We want to mention three synthesis papers related to the VLR and ZLC/IMR context. An outstanding synthesis paper by Hobbs and Aldhous [164] summarized earlier harvested data [136,165]. In that paper, the authors do not use any of these abbreviations showing their research neutrality. The data were collected using radar systems with a vertically pointing linearly polarised beam (with the rotating plane of polarization about the vertical axis at several Hertz offsetting from the vertical axis by about 10% of the beamwidth and scanned around the vertical at the same rate) and marine X-band transceivers. This work did not consider the wingbeat; the authors concluded that it could be a useful extension for the future. In 2004, the Australian group [166] provided detailed results on this topic (extending the previous finding on ZLC-radar presented in Reference [163]). They gathered wingbeat parameters using rotary-mode signals in an additional (final) stage of the data-processing procedure that routinely retrieves trajectory and target parameters from an IMR’s conical-scan observations detailed in the previous work. The English group presented their synthesis in Reference [162]. They discussed the insect aerial density’s vertical distribution in the atmosphere. The researchers described occasions during warm nights in the summer months when intense insect layers developed in the southern UK.

All these synthesis papers strictly positioned themselves regarding terminology, avoiding a “competing” research group’s abbreviations. After 2005, both research groups worked productively together. In Reference [167], they investigated the ventral-aspect radar cross sections of insects. Ventral means that an insect is oriented with its underside directed towards the beam. An insect data gathered with 9.4 GHz linear-polarization X band radar has been analyzed to identify relationships between RCS parameters and the insects’ masses and morphological dimensions and forms.

### 3.3. Low-Altitude Observations

All earlier discussed works belong to high-altitude flight observations using pulse radar. Excluding particular rare conditions, the ground clutter makes it impossible to detect low-altitude targets [168]. Harmonic radar resolves this problem using a diode reflector [169,170]. A tiny electronic diode (tag) glued to the tracking target is used for insect detection [171,172,173,174,175]; various tag designs have been developed [176,177].

In Reference [178], Psychoudakis et al. proposed a principally new type of transponder—a modified Minkowski loop tag. It consists of two concentric fractal loops for a radar unit transmitting a 5.9–6 GHz signal and detecting at the 11.8–12 GHz band. The proposed planar geometry (bendable) tag design allowed improving harmonic conversion efficiency; it had a smaller size (9.5 × 9.5 mm) than the earlier solutions and could detect a tagged insect up to 58 m. Even though the transponder was designed for insects, in that work, the authors, seemingly, did not test it with real insects and scheduled it for future work.

One can mention the importance of research disclosing the tags’ impact on the insect flight. Kim et al. addressed this issue in Reference [179]. The authors utilized a wire dipole tag described in References [180,181]: “the total length of the tag was 9 mm with a 1-mm-diameter loop at the pole and a 1 mm foot bent at 90${}^{\circ }$”. Kim et al. noticed the potential of the harmonic radar for three examined species; it showed a severe impact on two insects at rest.

Harmonic radar keeps attracting the height attention of researchers proposing various novel solutions. For instance, Hsu et al. [182] proposed the use of a pseudorandom code principle in harmonic radar to achieve high sensitivity. Advances in harmonic radar hardware and algorithms led to its in-production use for the insect behavior investigation. For instance, in Reference [183], researchers tracked many Chinese citrus flies for several years. They disclosed that early emerging adult insects migrate into forests. Kho et al. [184] investigated two methods for insect tracking in the grass field and confirmed the harmonic radar’s high efficiency. In Reference [185], Milanesio et al. presented a new harmonic radar prototype with a detection range of 500 m for locating Asian hornet nests. A solution for designing a 2.5/5 GHz harmonic radar for honey bee tracking was discussed in Reference [186]. Another design of a compact transponder for insect tracking was proposed in References [187,188]. Such works confirm the effectiveness of this technology.

### 3.4. Frequency Modulated Continuous Wave Radar

Most of the earlier mentioned works considered pulsed radar systems. As an alternative, frequency modulated continuous wave radar (FMCW) can be used for insect detection. FMCW radar is a popular technique for investigating layers in the atmosphere [189,190,191]. One of the earliest works on FMCW radar for insect detection was published in 1973 [192]. In Reference [193], Gallagher et al. detected meteorological echoes contaminated and obscured by echoes looking like the diurnal cycle of insect behavior. It has to be mentioned that FMCW sets have not reached wide attention before 2000. Thus, compared to pulsed sets, such systems have not been widely reviewed in the literature.

In Reference [194], Contreras and Frasier discussed the S-band FMCW mobile radar exploitation results for one month in Oklahoma. This design allowed the authors to reach an altitude of 2500 m. As in the work discussed in the previous paragraph, insects appeared as discrete dots in the resulting charts. The authors assumed that these dot echoes are insects (“assume to be insects”). Although they did not conduct experiments with artificially placed insects and other targets, their assumptions look convincing since it corresponds to independent findings in other works on insect radar. Furthermore, Contreras and Frasier proposed a two-dimensional (5 × 5) median filter to isolate insects’ contributions, which allowed for the distinguishing of target types.

In the last decade, several works proposed to apply FMCW radar to improve studies conducted with pulsed systems. In Reference [195], Tahir and Brooker proposed harmonic FMCW radar for tracking the insect. They noticed that, unlike pulse systems, FMCW radar does not require high peak power to be transmitted for a linear frequency chirp; it receives the second harmonic of a transmitted signal. Tahir and Brooker discussed UAV feasibility for harmonic radar operating in the millimeter-wave band where component size and weights are small. Tahir et al. continued the research in the next work [196], where they mainly discussed the design of new, very compact transponders. In that work, they did not have the goal to use FMCW radar with UAVs, but the authors showed the results of exploiting FMCW harmonic radar and millimeter wave harmonic sensors. They have noticed that “FMCW harmonic radar would meet the size and weight constraints for its fixation on a mobile vehicle or UAV for the continuous tracking of migrating insects fitted with harmonic sensors to beyond one km which is the maximum detection range achieved by existing pulse radar.” The idea of UAV-borne FMCW harmonic radar rose again in Reference [197], but as in the earlier discussed works, concrete prototypes have not been shown. An actual UAV aimed version of the harmonic radar is described in Reference [198].

The FMCW principle is used in different types of insect radar. Yang et al. have developed a VLR set using FMCW radar [199]. As discussed earlier, the pulsed X-band radar covered altitudes from 150 to 1200 m, while the FMCW system aimed at targets up to 200 m, i.e., filling the gap from the ground to 150 m. The device uses the same principle of observations as the pulsed VLR, i.e., the vertical-looking beam has an offset by a small angle around the vertical axis by aligning the upward-pointing vector. The insect detection results at 150 m height in the air look promising.

One can notice that FMCW systems are already utilized by vertical-looking and harmonic solutions. We expect attempts to develop scanning systems using FMCW radar in the future. Compactness and low energy consumption make such setups very promising.

### 3.5. Tendencies and Classification

**Figure 2:**
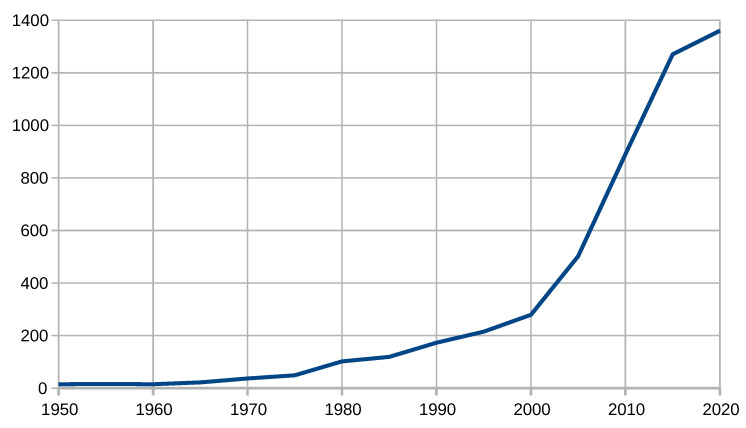
Number of publications on insect radar from 1946 to 2020 (five-year interval).

**Figure 3:**
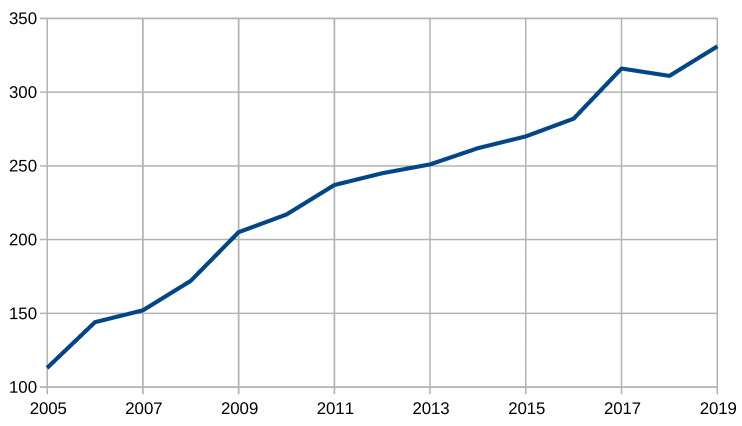
Number of publications on insect radar from 2000 to 2020 (one-year interval).

To illustrate the scientific interest in insect radar, we have summarized the number of relevant publications in time intervals. For this, we utilize the same approach used in Reference [200]. We queried Google Scholar with the following search phrase: (“*insect*” *or* “*entomology*”) *and* “*radar*”. Table 1 and Figure 2 show the number of publications aggregated in five-year intervals starting from 1946–1950 and ending in 2016–2020; Figure 3 shows the number of publications aggregated in a one-year interval starting from 2005.

According to Table 1 and the figures, insect radar is a well-developed methodology and an increasingly trending topic. It started from tens of publications a year and reached hundreds in the 1980s. In 2011–2015, it exceeded 1000, remaining far above this level. Of course, this process is also associated with a general trend of increasing the number of publications caused by the rise of the Internet. Figure 3 proves that this topic remains steadily growing because it shows the number of publications in recent years. It had been nearly linearly grown from 113 in 2005 to 331 in 2019. In 2020, 102 published items were already indexed.

In addition to radar research dynamics, it is necessary to figure out major radar types, groups, and classification. In Reference [95], Drake and Reynolds considered radar entomology from multiple viewpoints. They have distinguished radar sets by their configuration, observation type, and applications. For instance, short-, medium-, and long-range entomological radars are recognized by transmission modes. Regarding the configuration, scanning, profiling, and airborne radar sets are distinguished. In Reference [201], the authors recognized the following radar types: airborne (AER), scanning (SER), tracking (TER), and vertical-looking (VLR) entomological radar systems. In 2004, Chapman, Reynolds, and Smith, in their review work [202], discussed two main groups of insect radar: vertical-looking and tracking radar; tracking radar in this subdivision consisted of scanning and harmonic systems. Later, in 2011, Chapman, Drake, and Reynolds [159] refined their earlier classification by distinguishing three main insect radar groups: scanning, vertical-looking, and harmonic; moreover, they acknowledged the potential of FMCW systems.

We want to propose a simple classification of insect radar sets based on the last-mentioned publications, taking into account the radar working principle. The two following major radar groups are discussed in multiple references: pulsed and FMCW solutions. These groups are imbalanced; much more work has been done on the pulsed radar. FMCW is likely to be the leading solution in the future due to its compactness and cost. We want to note that radar satellite imagery [203] and ground-penetrating radar [204] are out of the scope in this classification (but both have a high potential for insect monitoring) because such solutions observe insects indirectly (e.g., through canopy properties, insect tunnels, etc.).

**Figure 4:**
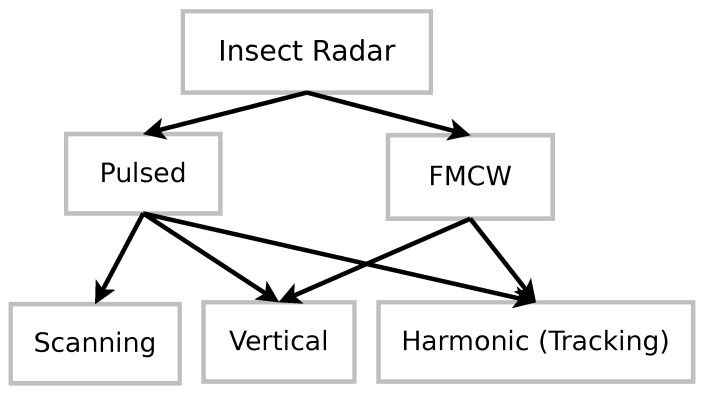
Insect radar groups.

We propose a two-level classification of insect radar systems using the discussed ideas. It is altered from the previous descriptions by acknowledging recent work on FMCW systems. Our updated classification is reflected in Table 2 and Figure 4.

Scanning radar systems carry out small-scale observations and cover large areas. They were under active development in the early stage of insect radar. Later, vertical-looking approaches have become more popular because they allowed researchers to increase the study scale and gather better-granulated data. Recently, harmonic radar enabling large-scale research has become popular (but it can track only a single (or a few) insects equipped with a tag, i.e., not suitable for mass monitoring). FMCW radar applications are becoming very promising due to their compactness and low-energy consumption. Even though we have not found any papers concerning the use of FMCW in a scanning mode, we can admit that it is technically possible. Thus, one can see it as a significant gap and a good method for new low-range radar prototypes. Such low-range systems can be utilized, for example, for pest management in open agricultural fields.

The FMCW principle allows small range observations. Further, we discuss some novel ideas and solutions on insect radar derived from the provided literature review. In the context of our future work, we consider the only compact small-range and energy-effective solutions. One can point out that only the FMCW radar can meet our expectations.

To describe dynamics in insect radar research, we have prepared Table 3. It shows the number of publications mentioning insect radar groups published in the two following time intervals: all-time and a time interval since 2015. Moreover, the percentage of papers published since 2015 is provided in the last rows of all sub-tables. We want to emphasize the disproportion of the distinguished radar groups reflected by tables.

The lowest sub-table shows the number of publications mentioning radars of different radar frequency bands [120], confirming the growing interest in compact low-range systems. One can expect improvements in balancing the discussed insect radar groups due to technical progress, advances in big data processing and machine learning, and the growing demand for compact and resource-effective solutions that increase the popularity of FMCW systems.

## 4. Future Work

Insect radar systems are becoming compact. Some authors have even mentioned their intention to install novel compact radar devices on UAVs. We have noted a definite gap in radar entomology: ranges from 0 to tens of meters are not covered with the existing systems.

**Figure 5:**
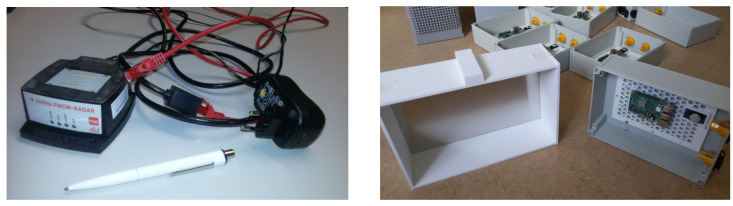
(**left**) a radar device; (**right**) constructing light traps (work in progress).

**Figure 6:**
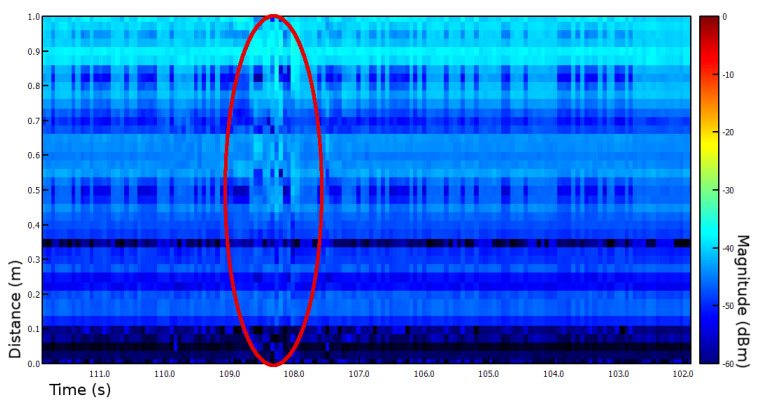
History plot showing an insect (108–109 s).

Assembling both these findings, we have come up with an idea of two novel insect radar setups. Due to a small range, it is problematic to encounter the significant insects’ biomass. Thus, for both setups, we use light traps. Moreover, for collecting ground truth data, we intend to use cameras. Figure 5 left presents a 24 GHz FMCW radar device that we use for both setups; preliminary estimations showed that insects could be detected with this device. For instance, Figure 6 shows a time plot with the registered insect presence.

The first setup (see Figure 5) has a typical modern design consisting of an attracting screen and a computing box with a camera. A camera connected to a Raspberry Pi computer aims to a screen and uses computer vision algorithms to detect insects. The attracting screen consists of an aluminum sheet, light diffuser, and UV lights. Radar should be installed between a camera box and the attracting screen. Thus, it can detect insects flying towards the light. Radar does not play a key role in this configuration; it accompanies the camera.

**Figure 7:**
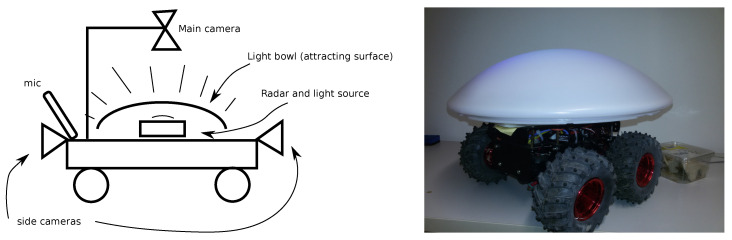
Insect radar and rover: (**left**) a concept; (**right**) a mockup installation with a rover, radar, and light trap.

The presented setup attracts the insect from the limited volume. Moreover, it is static, aiming to be used in one place. To overcome these limitations, we propose a light trap with radar installed on an unmanned ground vehicle (UGV, or rover). In such a setup, radar plays a central role. Figure 7 illustrates our idea, i.e., installing radar surrounded with UV light sources in the middle of the rover and covering it with a light diffuser. Since the diffuser has a bowl shape, it can attract insects from a maximum volume. The night camera is pointed at the light trap, while the day camera is pointed to the sky; this allows full-day insect species tracking and detection. A key point in such an installation is radar. It will be capable of estimating the insect biomass in a large volume around the setup. Assembling radar and camera data will make it possible to collect information regarding insect species, biomass, and dynamics. Prospectively, the rover can be programmed to drive autonomously in the forest during the night along predefined paths. It will be equipped with a camera to collect ground truth data. Additional sensors can facilitate other purposes of the rover.

Preliminary tests showed that these setups have high potential and should be developed further. Currently, only insect presence and biomass can be investigated. Due to technological limitations, it is impossible to detect discrete insect targets. We can see insects only as dynamic noise, which is why the camera plays a vital role in collecting ground truth data.

**Figure 8:**
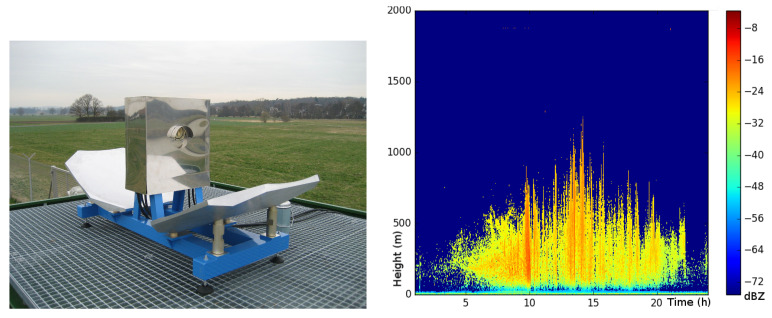
(**left**) operating 94 GHz frequency-modulated continuous-wave (FMCW) cloud radar; (**right**) diurnal bioaerosol.

The described 24 GHz radar device was designed to detect targets up to 20 m from the radar. We can observe higher insect targets with another obtained instrument. Our laboratory operates 94 GHz FMCW cloud radar (see Figure 8 left) for meteorological purposes [207]. The radar site is located in Marburg Ground Truth and profiling station in Linden-Leihgestern, Germany (50.533N, 8.685E, 172 AMSL). This radar provides a 10 m spatial and a 10 s temporal resolution of cloud structure [208]. The radar can detect clouds as low as 30 m [209]; it can detect targets to a height of at least 8 km.

It is well known that such systems’ data comprise a large amount of insect clutter [210]. Researchers often describe this phenomenon as “bioaerosol” because it is a mixture of insects and other bio-materials, such as pollen. Figure 8 right shows a profile of cloud radar reflectivity for a cloud-free day. It illustrates a diurnal course of a signal, which culminates around noon. This means that the “bioaerosol” is extended by increasing thermal turbulence (the boundary layer is extending) and because the insects can fly higher when it is warmer. While Luke et al. [210] have considered such a signal as a contaminant and have tried to filter out it, this is target information for our future work.

Combining the described radar systems would allow us to conduct systematic multi-scale insect monitoring. 24 GHz radar equipped with light trap allows low-altitude insect monitoring. 94 GHz cloud radar would aim for high-altitude insect flight information gathering. Since both systems are compact and relatively inexpensive, prospectively, this combination can be used to deploy network stations for insect monitoring. Moreover, the setup with a rover shows a good perspective to collect large-scale spatial information of the insect distribution.

## 5. Conclusions

The present article provides a review of works that show the potential to find a solution for systematic and global insect monitoring to respond to the “Ecological Armageddon” problem (discussed in the introduction). We indicated that insect traps and remote sensing solutions are the two primary monitoring methods. In this work, we have discussed essential approaches to insect monitoring.

Firstly, sampling methods comprising mainly various insect traps have been discussed. Secondly, remote sensing approaches allowing non-intrusive insect monitoring have been considered. It has been indicated that compaction and cost-effectiveness allow for the wide use of remote sensing methods for insect monitoring. Recent solutions utilize various cameras, UAVs, lidar, and other modern achievements.

Radar remains the most popular technique among remote sensing solutions. Pulsed-scanning, vertical-looking, harmonic, and frequency-modulated continuous-wave (FMCW) radar solutions are considered. Pulsed-scanning became popular in the 1980s; it covers large areas around the setup, which is useful for massive insect migration observations. Vertical-looking systems facilitate the collection of very detailed information about high-altitude insect flights. Widespread harmonic radar is being designed to track individual insects and work at low altitudes. Recent achievements in FMCW radar technology allow for the production of compact devices, making it an up-and-coming system.

We have indicated a continually growing interest in insect radar, and it has been confirmed by the number of relevant papers published since the 1950s. Since 2005, the growth has remained linear. We expect that the interests will be much higher due to recent technology achievements. Real-time and big data analytics, machine learning, compact and low-cost sensors, and embedded systems support this growth today. Blockchain and decentralized solutions are leveraging it in the near future.

Additionally, we have proposed an updated classification of insect radar systems. Two large groups are pulsed and FMCW sets. Pulsed can be scanning, vertically oriented, or harmonic. Vertically oriented and harmonic sets can also be considered a subset of FMCW. The provided relevant references for each group illustrate this classification.

We have proposed novel radar setups with an FMCW radar system for the future. Since the utilized radar is very compact, we will install it on a rover equipped with multiple sensors and a light trap. We intend to run the rover autonomously in the forest to collect comprehensive and continuous information about insects. Furthermore, we plan to develop a static setup with radar and an attractive-screen-based light trap. Finally, to gather multi-scale data, we will use 94 GHz cloud radar to observe insects at high altitudes.

We believe that the insect monitoring expectations discussed in the introduction can be addressed in the near future. Today, the market proposes compact and cost-effective solutions; with big data and machine learning achievements, monitoring can be organized globally, continuously, and systematically. Intensive and collaborative work is required for successful progress.

## Figures and Tables

**Table 1:** Number of publications on insect radar according to Google Scholar.

Years (1946–2020)	Amount	Years (2005–2019)	Amount
1946–1950	15	2005	113
1951–1955	16	2006	144
1956–1960	15	2007	152
1961–1965	22	2008	172
1966–1970	37	2009	205
1971–1975	49	2010	217
1976–1980	102	2011	237
1981–1985	119	2012	245
1986–1990	173	2013	251
1991–1995	215	2014	262
1996–2000	279	2015	270
2001–2005	502	2016	282
2006–2010	890	2017	316
2011–2015	1270	2018	311
2016–2020	1360	2019	331

**Table 2:** Insect radar groups and relevant sample publications (not a complete list).

Radar Orientation	Pulsed	FMCW
High-Altitude: Scanning Large Areas	[90,93,137,138,139,140,141]	
High-Altitude: Vertically Oriented (including VLR)	[135,150,151,157,158,160,163,164,166]	[192,193,194,199,205,206]
Low-Altitude: Harmonic	[169,172,173,174,175,176,177,178,179,180,181,183]	[178,182,195,196,197]

**Table 3:** Estimated numbers of publication mentioning relevant insect radar groups.

	Pulsed	FMCW	Scanning	Vertical	Harmonic	C-Band	S-Band	X-Band	Ku-Band	Ka-Band
All-time	5100	50	29,900	500	2890	440	210	290	20	30
Since 2015	1240	20	8331	140	1010	110	80	90	10	10
% since 2015	24	40	28	28	35	25	38	31	50	33

## Data Availability

Not applicable.
